# Disease, activity and schoolchildren’s health (DASH) in Port Elizabeth, South Africa: a study protocol

**DOI:** 10.1186/s12889-015-2636-y

**Published:** 2015-12-23

**Authors:** Peiling Yap, Ivan Müller, Cheryl Walter, Harald Seelig, Markus Gerber, Peter Steinmann, Bruce P. Damons, Danielle Smith, Stefanie Gall, Dominique Bänninger, Thomas Hager, Nan S. N. Htun, Liana Steenkamp, Annelie Gresse, Nicole Probst-Hensch, Jürg Utzinger, Rosa Du Randt, Uwe Pühse

**Affiliations:** Department of Epidemiology and Public Health, Swiss Tropical and Public Health Institute, P.O. Box, CH-4002 Basel, Switzerland; University of Basel, P.O. Box, CH-4001 Basel, Switzerland; Department of Sport, Exercise and Health, University of Basel, St. Jakobsturm, Birsstrasse 320B, CH-4056 Basel, Switzerland; Department of Human Movement Science, Nelson Mandela Metropolitan University, P.O. Box 77000, Port Elizabeth, 6031 South Africa; Department of Dietetics, Nelson Mandela Metropolitan University, P.O. Box 77000, Port Elizabeth, 6031 South Africa; Sapphire Road Primary School, P.O. Box, Booysens Park, Port Elizabeth, 6059 South Africa

**Keywords:** Anthropometry, Cognitive performance, Diabetes, Health interventions, Intestinal parasite infections, Physical fitness, Physical activity, Psychosocial health, South Africa

## Abstract

**Background:**

An in-depth epidemiological investigation on intestinal parasite infections in an impoverished area of Port Elizabeth, South Africa provides a unique opportunity for research on its impact on children’s physical fitness, cognitive performance and psychosocial health. Additionally, we will screen risk factors for the development of diabetes and hypertension in adulthood.

**Methods/Design:**

A 2-year longitudinal cohort study will be conducted, consisting of three cross-sectional surveys (baseline and two follow-ups), in eight historically black and coloured (mixed race) primary schools located in different townships in Port Elizabeth, South Africa. Approximately 1000 Grade 4 primary schoolchildren, aged 8 to 12 years, will be enrolled and followed. At each survey, disease status, anthropometry and levels of physical fitness, cognitive performance and psychosocial health will be assessed. After each survey, individuals diagnosed with parasitic worm infections will be treated with anthelminthic drugs, while children with other infections will be referred to local clinics. Based on baseline results, interventions will be tailored to the local settings, embedded within the study and implemented in half of the schools, while the remaining schools will serve as controls. Implementation of the interventions will take place over two 8-week periods. The effect of interventions will be determined with predefined health parameters.

**Discussion:**

This study will shed new light on the health burden incurred by children in deprived urban settings of South Africa and provide guidance for specific health interventions. Challenges foreseen in the conduct of this study include: (i) difficulty in obtaining written informed consent from parents/guardians; (ii) administration of questionnaires in schools where three languages are spoken (Afrikaans, Xhosa and English); (iii) challenges in grasping concepts of psychosocial health among schoolchildren using a questionnaire; and (iv) loss to follow-up due to the study setting where illiteracy, mobility and violence are common. Finally, designing the health interventions together with local principals and teachers will allow all concerned with the research to bolster a sense of community ownership and sustained use of the interventions after the study has ceased.

**Trial registration:**

Controlled-trials.com; identifier: ISRCTN68411960 (date assigned: 14 February 2014).

## Background

As traditional lifestyle and diet change alongside socioeconomic developments, countries are starting to experience a double burden of communicable and non-communicable diseases in the face of weak health systems [1, 2]. Many countries still struggle to meet the existing challenges stemming from infectious diseases, such as malaria and intestinal parasite infections. Meanwhile, non-communicable diseases, such as diabetes, cardiovascular diseases, obesity-related conditions and cancers, impose a growing burden on them [3]. This phenomenon has been recognised by the global health community and must be addressed in the new era of the sustainable development goals (SDGs), particularly “to ensure healthy lives and promote well-being for all at all ages” [4], while the unfinished agenda of the communicable diseases during the millennium development goal (MDG) era must be accelerated.

In South Africa, investigations of physical activity patterns of primary schoolchildren attending schools in disadvantaged neighbourhoods have confirmed that physical activity levels are insufficient [5]. These school environments are usually not conducive for the promotion of physical activity due to inadequate sport and recreation facilities, a lack of qualified teachers and an irregular physical education program. In 2010, Kimani-Murage *et al.* [6] reported that in a low-income South African setting, the co-prevalence of early stunting and adolescent obesity in girls is a result of increasing levels of physical inactivity. This observation was particularly prevalent among black girls, who were found to have the highest rates of physical inactivity [7]. As physical inactivity during childhood can lead to poor health outcomes in adulthood [8], there is a pressing need to promote physical activity among school-aged children in disadvantaged communities in order to prevent obesity-related conditions and other non-communicable diseases. Additionally, infectious diseases that are intimately connected with poverty may also occur in disadvantaged South African schools [9]. These infections can have a negative impact on children’s nutritional status, cognitive abilities and physical fitness [10, 11]. Such a dual burden of disease can put children at a high risk of compromised health, poor subjective well-being, hampering their growth and economic perspectives.

**Fig. 1:**
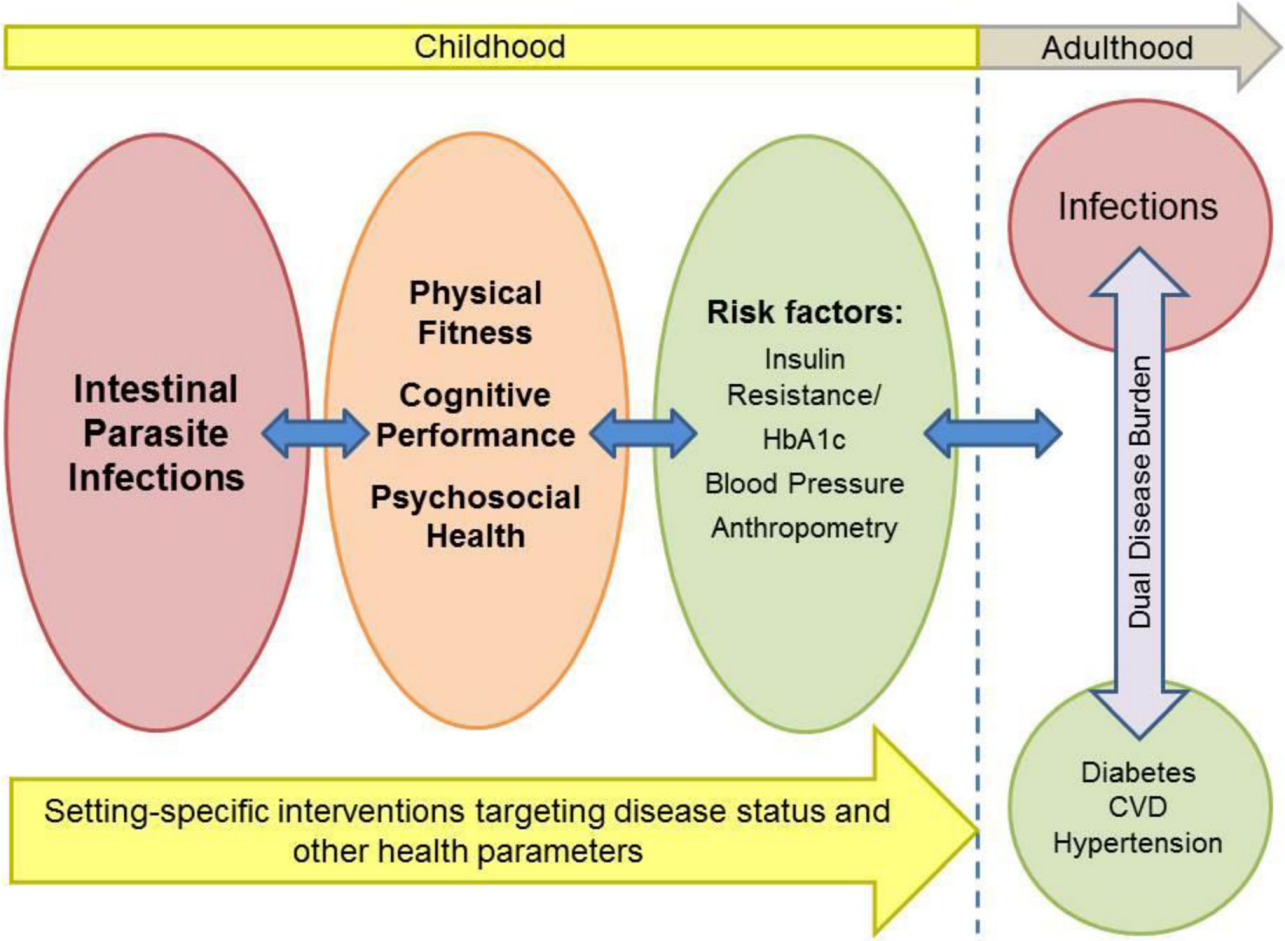
A conceptual framework for the DASH study

In particular, it is hypothesized that, first, intestinal parasite infections have a negative influence on the physical fitness, cognitive performance, nutritional status and psychosocial health of school-aged children in deprived urban South Africa. Second, the development of setting-specific health interventions can decrease the incidence of parasitic infections and insulin resistance as well as elevated blood pressure, and thereby the risk of developing non-communicable conditions later in life, such as diabetes and hypertension (Fig. 1). An in-depth epidemiological study on intestinal parasite infections in an impoverished part of Port Elizabeth, South Africa, will provide a unique opportunity for research on its impact on children’s physical fitness, cognitive performance and psychosocial health. In addition, a search for risk factors for the development of diabetes and hypertension in adulthood seems justified. From this research, an evidence-base will be created to design setting-specific health interventions. The purpose of this article is to present the detailed protocol of the proposed study.

### Goal and objectives

The goal of this project is to survey the distribution of selected intestinal parasite infections and risk factors for non-communicable conditions, and assess their impact on schoolchildren’s health over time in the face of tailored interventions in eight townships of Port Elizabeth, South Africa before, during and after the introduction of setting-specific interventions. We will pursue two specific objectives:

(i) to conduct a longitudinal study assessing the prevalence of intestinal parasites, risk factors for diabetes and hypertension, anthropometry and the level of physical fitness, cognitive performance and psychosocial health among schoolchildren; and
(ii) to design setting-specific interventions and assess their effect on the measured health parameters.

## Methods/Design

### Study area

**Fig. 2:**
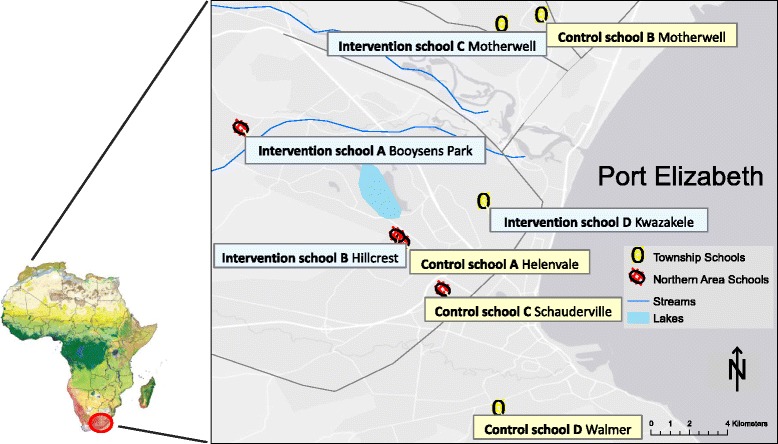
Study area and location of schools participating in the DASH study

The study will be conducted in historically black and coloured (mixed race) government primary schools from various areas in Port Elizabeth in the south-east part of South Africa (Fig. 2). The areas populated by black Africans are commonly referred to as townships and include the areas of Kwazakhele, New Brighton, Zwide, and Motherwell. The “Northern areas” in Port Elizabeth are largely made up of coloured people who were forcefully relocated from the central areas of the city to the outlying northern areas, and include the areas of Schauderville, Gelvandale, Helenvale, Hillcrest and Booysens Park [12, 13]. Government schools in South Africa are classified into 5 groups, called quintiles, mainly for the purpose of allocating financial resources, with quintile 1 being the poorest and quintile 5 being the “least poor”. Schools in quintiles 1, 2 and 3 are proclaimed as no-fee schools, while schools in quintiles 4 and 5 are fee-paying schools [14]. The eight schools who will be participating in the DASH study belong to quintiles 3. Furthermore, the study area has been detrimentally affected by extreme poverty and high rates of unemployment, due to past government policies, as well as current public health and economic challenges faced by the country [7].

### Study design

**Fig. 3:**
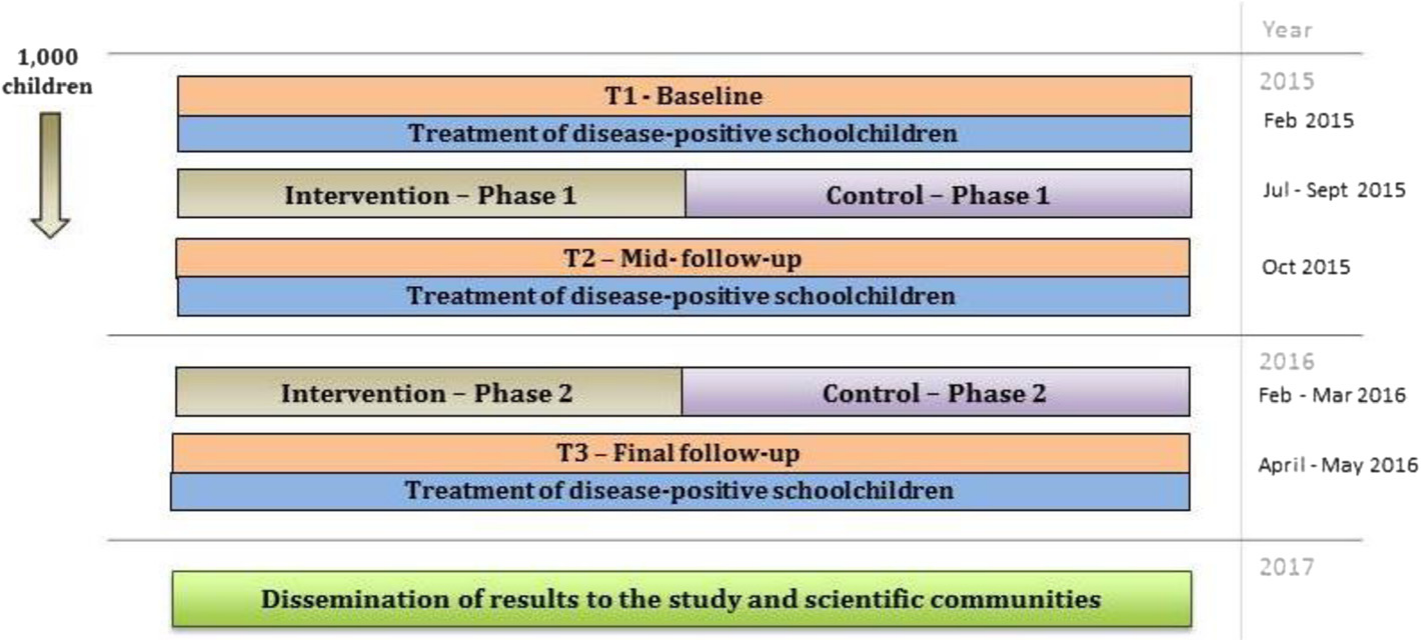
A pictorial display of the design and timeline of the DASH study

The study duration spans from February, 2015 to June, 2017 (Fig. 3). The longitudinal cohort study consists of three cross-sectional surveys (baseline, mid- and final follow-up). At each survey time point, disease status, anthropometry and levels of physical fitness, cognitive performance and psychosocial health are measured. After each survey, infected individuals are either treated with anthelminthics (400 mg albendazole, single dose) for soil-transmitted helminths [15] and/or referred to local clinics for the management of other intestinal parasite infections. Based on results from the baseline survey, a package of setting-specific interventions is designed together with local students, teachers, school volunteers and parents. The intervention package consists of three main components:

(i) Physical activity [9, 16]: Regular physical activity opportunities, including two physical education (PE) lessons a week, weekly dancing-to-music classes, and in-class activity breaks will be incorporated into the main school curriculum and a physical activity friendly school environment will be created. These approaches could help improve children’s physical fitness, and positively affect their psychosocial health [17–21].
(ii) Health education [22]: A series of classroom-based lessons will be developed to help increase the awareness for intestinal parasite infections among the schoolchildren and educate them on treatment and prevention methods, such as proper hygiene, sanitation habits and the importance of consuming clean water and food. It is also planned that schoolchildren will produce a theatre play to convey key messages they have learnt through the health education.
(iii) Nutritional interventions: A series of classroom-based lessons will be developed to help increase the awareness of the importance of healthy nutrition. In addition, an analysis of the school feeding programme will be done to identify ways to improve their current diet to be healthier. The schoolchildren will also be given a ready to use supplementary food (RUSF) in the form of an enriched lipid-based paste. The cooks in the schools will also be trained in basic nutrition and hygiene during preparation of the school meals.

The interventions will be embedded within the longitudinal study and be implemented in half of the schools, while the remaining schools will serve as controls. The intervention schools were either selected due to the soil-transmitted helminth prevalence, number of Grade 4 learners, geographical location and affiliation to a particular ethnic group or commitment of teachers or school staff. Implementation of the interventions will take place twice; in July-September, 2015, after the baseline survey, and in February-March, 2016, after the first follow-up. The first follow-up will allow the implementation feasibility of the designed interventions to be determined through focus-group discussions with teachers and students, while the subsequent surveys will allow assessing their impact on the measured health parameters.

### Sample size

The sample size calculation for the study was based on achieving sufficient precision in estimating the prevalence of soil-transmitted helminth infections. We conducted our calculation under the following assumptions for the cross-sectional baseline study:

i) a prevalence of soil-transmitted helminth infections, *p*, of approximately 3 %;
ii) an average number of children per school, *B*, of 150; and
iii) an intra-class correlation coefficient for the clustering of outcomes within schools, *ICC*, of 0.15.

Requiring the standard error of the respective prevalence, *SE*, not exceeding 2.5 %, we obtained a necessary sample size *n* of 1088 children, using the formula [23]

$$ n\kern0.5em \cdot \ge \frac{p\cdot \left(1-p\right)}{S{E}^2}\left(1+\left(B-1\right)\cdot ICC\right) $$

As a consequence, eight clusters (schools) will be needed considering the fact, that with a total of 1200 children from eight schools, we can accommodate 10 % loss to follow-up.

### Study participants

Children will be invited to participate if they meet the following inclusion criteria: (i) are willing to participate in the study; (ii) have a written informed consent by a parent/guardian; (iii) are not participating in other clinical trials during the study period; and (iv) do not suffer from medical conditions, which will prevent participation in the study, as determined by qualified medical personnel. Approximately 1000 Grade 4 primary schoolchildren, aged around 8 to 12 years, from 8 schools will be recruited during the baseline survey.

### School selection, participant recruitment and written informed consent

School authorities will be briefed about the project and their approval sought. Subsequently, a description of the project will be delivered by hand to 103 government primary schools and principals will be encouraged to allow their schools to participate in the study. Those schools with positive written responses will be invited to a comprehensive information meeting with the study investigators. Thereafter, interested schools will be visited and the study investigators will talk to the school management from these schools to find out whether the school environment is conducive for the performance of the study. Selection of schools will be based on size of the Grade 4 classes (*n* > 100), geographical location and population demographics (Xhosa-, Afrikaans- and English-speaking schoolchildren). School principals and teachers of the selected schools will be notified about the study aims, procedures and potential risks and benefits. Schoolchildren, parents or legal guardians of learners will then be informed and schoolchildren encouraged to participate in the study. Before the launch of the study, a patient information sheet in English, including translation into the local language (Xhosa or Afrikaans), will be given to all potential participants and their parents/guardians, explaining the objectives, procedures and potential risks and benefits of the study. The name and contact address of the main investigator on site will be provided for any specific follow-up question. Oral assent from each participating schoolchild will be sought, while individual written informed consent will be required from parents/guardians. For illiterate parents, the information sheet will be read aloud and, if need be, an oral translation of the information sheet into all the local languages (Xhosa, Afrikaans or English) will be given. Participation is voluntary, and hence, children can withdraw from the study at any time without consequences and further obligation. Finally, demographic data and socioeconomic status of each participant will be obtained via the use of a questionnaire.

### Assessment methods

**Fig. 4:**
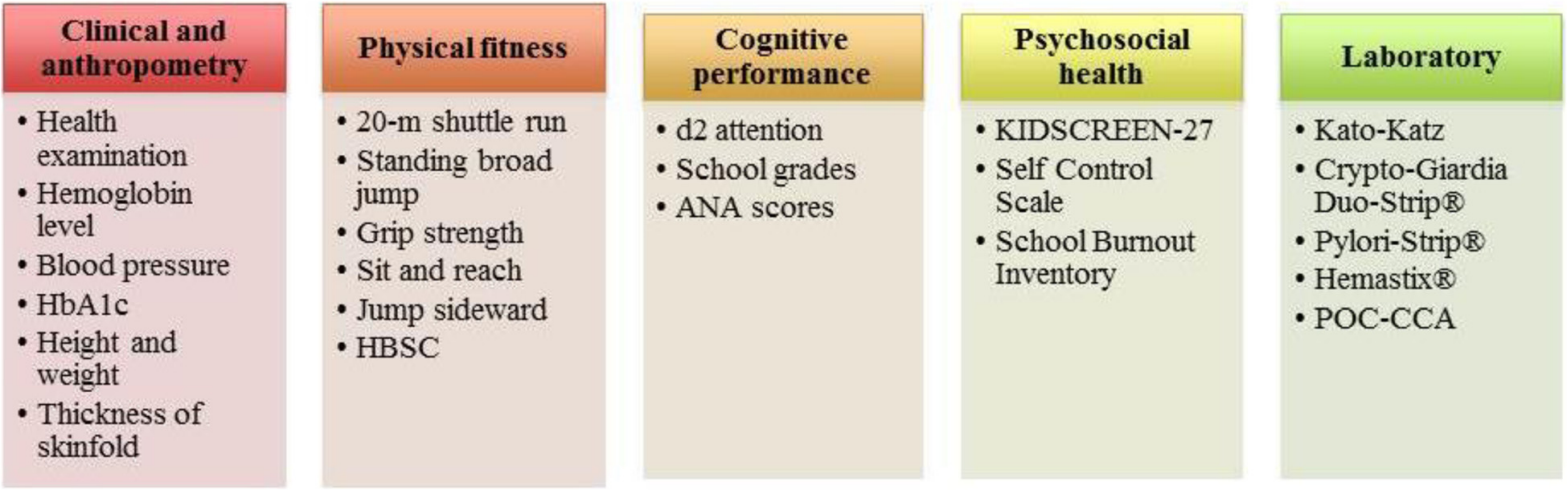
A summary of the measurements and tests performed under the DASH study

Figure 4 summarises the assessment methods to be used in this study. For each cross-sectional survey, a specific combination of the following procedures will be selected and conducted by well-trained staff, adhering to standardised and quality-controlled protocol.

#### Health examinations

(i) Clinical examination of children will include detailed medical history taking and physical examination. Features of patient history will focus on fevers, constitutional symptoms, abdominal pain and change in bowel movements. Physical examination is directed towards evidence of anaemia (e.g. conjunctival pallor), detailed abdominal examination (e.g. tenderness, hepatomegaly and splenomegaly) and evidence of pulmonary hypertension (e.g. jugulovenous pressure and cardiac auscultation).
(ii) Four questions about food security in the past two days, based on a simplified version of the Household Food Insecurity Access Scale (HFIAS) will be asked [24].
(iii) For the detection of anaemia, the hemoglobin concentration will be measured once (to the nearest 0.1 g/l) using a HemoCue® Hb 301 system (HemoCue®AB; Ängelholm, Sweden). A fresh set of alcohol swab, safety lancet and microcuvette will be used for each child. After swabbing the fingertip with alcohol, the field investigator will prick it with a safety lancet and squeeze gently for two drops of blood. The first drop will be wiped away with the alcohol swab, while the second drop will be taken up by the microcuvette and read by the device. The Eurotrol Hb 301 Control will be used to verify the precision and accuracy of the measuring system (Since there will be other tests, which involve the use of whole blood from a finger prick, we will ensure an organised procedure so that each child is only pricked once.)
(iv) For the measurement of blood pressure, each child’s blood pressure will be measured once with the Omron® digital blood pressure monitor, while the child is seated. The cuffs will be wrapped around the left arm such that one finger could fit between the cuff and the arm. The bottom of the cuff is about 4.0 cm above the elbow and the palm should be facing up while blood pressure is being measured.
(v) For the measurement of glycated haemoglobin (HbA1c) level, a point-of-care instrument employing the Afinion test (Alere Technologies) will be used. This test is based on boronate affinity separation and the use of fluorescence quenching, with results available in about 3 min. This method meets the generally accepted performance criteria for HbA1c as defined by the National Glycohemoglobin Standardization Program (NGSP) and has no interference from HbC, HbS, HbE and HbD traits results. Of note, the HbA1c level reflects the average plasma glucose concentration levels over the previous 8–12 weeks before measurement with no prior fasting required. A fresh set of alcohol swab, safety lancet and HbA1c test cartridge will be used for each child. After swabbing the fingertip with alcohol, the field investigator will prick it with a safety lancet and squeeze gently for two drops of blood. The first drop will be wiped away with the alcohol swab, while the second drop will be taken up by the test cartridge and read by the Alere Afinion AS 100 Analyzer. Identical Afinion HbA1 control blood will be used as an internal control and tested at regular intervals to control for potential laboratory drift.

#### Anthropometric measurements

(i) Each schoolchild will be asked to take off their shoes and sweater before standing on the digital weighing scale. Body weight will be measured once and recorded to the nearest 0.1 kg.
(ii) With the shoes off, each child will stand against a stadiometer with their back erect and shoulders relaxed. Body height will be measured once and recorded to the nearest 0.5 cm.
(iii) Body mass index (BMI) and two specific Z-scores will be calculated, as follows: (i) BMI = weight (kg) / (standing height [meters (m)]^2^); (ii) BMI-for-age (BMIZ); an indicator for weight-for-height proportion (WHO growth reference for children older than 60 months) [25]; (iii) Height-for-age (HAZ); an indicator for stunting (WHO growth reference for children older than 60 months).
(iv) The thickness of the skinfold will be measured at two sites, namely triceps and subscapular [26, 27]. Before measurement, the field investigator will show the Harpenden skinfold caliper to the child and clamp it normally on the child’s finger to show that the process will not hurt. During the measurement, the child will stand with arms and shoulders relaxed. With the thumb and forefinger, the field investigator will gently pinch the skin (a vertical skinfold) slightly above the middle of the back of the arm (triceps) and clip the caliper (mouth of caliper is perpendicular to skinfold). After counting for 2 s, the reading should stabilize, and hence, it will be recorded. The field investigator will release the pinch but let the fingers stay in the same position on the arm and repeat the measurements two additional times. The three values obtained should be no more than ±5 % different from each other. If this is not the case, the measurements will be repeated. The final reading is an average of the three values. The same procedure applies for the subscapular site directly underneath the shoulder blade.

#### Physical fitness tests and self-reported physical activity

Previous studies in South Africa have used the Eurofit fitness testing battery [28]. For the purpose of this project, specific tests from the Eurofit fitness testing battery will be conducted in outdoor settings.

(i) The children’s cardiorespiratory fitness will be measured with the 20-m shuttle run test [29]. In brief, a 20-m flat running course will be measured with a measuring tape and marked with cones. Ten running lanes will be designated. Before the start of the test, children will be told to indicate any body discomfort and anyone who feels sick or not comfortable will not take part in the test. The pre-recorded sound signals will be played to the children and they will be initiated to do trial run of two intervals (2 × 20 m). Once children are familiar with the test procedures, they will be asked to run, in groups of five or ten, back and forth on the 20 m flat course by following the pre-set pace of sound signals. Starting with a running speed of 8.5 km/h, the frequency of the signal will be gradually increased so that every minute, the pace increases by 0.5 km/h. When children fail to follow the pace in two consecutive intervals, they will be asked to stop and the stage and the distance completed (full laps) will be recorded. The age of the participating child and the speed at which the child stopped running will be converted into VO_2_ max estimates, which is the maximum volume of oxygen that can be utilized within 1 min during exhaustive exercise, with an equation put forth by Léger *et al*. [29].
(ii) Lower body strength will be estimated with the standing broad jump test. Before the start of the test, the field investigator will demonstrate how exactly the standing broad jump is performed. Each child will stand behind a straight line and then jump as far forward as possible with both legs. Children will have 2 tries (with a 30 s rest in between) and the longest jump will be recorded (to the nearest 1 cm). The distance of the jump is measured from the starting line to the heel of the most back foot.
(iii) Upper body strength will be determined with the grip strength test. The TKK® dynamometer will be used for this test. Before the start of the test, the hand span (distance from the tip of the thumb to the tip of the little finger) of the child’s dominant hand will be measured (to the nearest 0.5 cm) and the grip span on the dynamometer adjusted accordingly [30, 31]. The field investigator also demonstrates how to grip the dynamometer to the child. Each child will have two tries (with a 30 s rest in between) to grip the dynamometer as hard as possible with both hands and the maximum readings (measured to the nearest 0.5 kg) will be recorded. Additionally, the dominant hand will be noted.
(iv) The Sit-and-Reach Test (SRT) will be conducted as an indication of flexibility. This test measures flexibility of the hamstring muscles (back of the thigh) and, to a minor extent, the lower back muscles. The study participant will be asked to sit on the floor with stretched legs and feet against the sit-and-reach box. The hands are placed over each other and with the hips bent forward as far as possible, the fingers should move as far as possible to the front. The distance between the fingertips and the back edge of the box will be measured.
(v) For the measurement of coordination skills and speed strength of the leg muscles, we will use the jump-sideward test. The task for the participant is to jump laterally with both legs at the same time as many times as possible within 15 s across a wooden bar. A field investigator demonstrates the test in advance and five jumps can be practiced.
(vi) The children will be asked questions about experiencing physical activity, such as doing sports, specific activities during school, playing with friends in their free time and walking to school. A recall period of 7 days will be used. The questions will be adapted from the Health Behaviours in School Age Children Survey (HBSC), an instrument used to gain insight into young people’s well-being, health behaviours and their social context [32].

#### Cognitive performance

Three measures will be considered as indicators of cognitive and academic performance, namely a standardized attention test (d2), children’s school grades, and the results of standardized national tests (ANA).

(i) The d2 test will be employed to measure attention performance. This test is among the most widely used measures of attention, particularly visual attention, in Europe and the USA [33]. The d2 paper-and-pencil version, which can be performed in a group setting, assesses several dimensions of cognitive performance: (i) total number of items processed (TN), a highly reliable measure of processing speed; (ii) percentage of errors (E%), measuring the qualitative aspects of performance; and (iii) the total number of items processed minus errors (TN-E), as an indication of the implications of the combined speed and accuracy scores for attentional and inhibitory control. Criterion, construct and predictive validity of the d2 test among children aged 9 years and above are well documented [34–36]. Moreover, the test offers an extensive list of norms, according to age, sex and education.
(ii) In cooperation with the schools, we will obtain school test grades from the following subjects: English, mathematics, home language and life orientation. The sum-score of the four subjects will be used to estimate academic achievement.
(iii) The Annual National Assessments (ANA) are standardised tests for literacy and numeracy in the foundation phase (Grades 1–3) and Mathematics and languages in the intermediate phase (Grades 4–6). For the purpose of our study, Mathematics and home language ANA scores will also be used as a measure for academic achievement.

#### Questionnaires for assessment of psychosocial health

To assess children’s psychosocial health, the following paper-and-pencil questionnaires will be applied:

(i) The KIDSCREEN-27 will be used to assess children’s physical and psychological well-being, moods and emotions, self-perception, autonomy, parent relation and home life, financial resources, peers and social support, school environment and bullying. The questionnaire includes 27 items and has been proven to be a valid instrument to assess psychosocial health of children aged 8–18 years across various countries [37–39].
(ii) Six items of the short version of the Self-Control Scale (SCS) will be used to assess individual differences in the capacity for self-control [40]. The human capacity of self-control has been described as one of the most powerful and beneficial adaptations of the human psyche [19, 40–43]. The exertion of self-control strengthens the relationship between the self and the environment, which is an important prerequisite for individuals’ satisfaction with life, well-being and positive development [44, 45]. Evidence for the reliability and validity of the SCS has been demonstrated previously [40].
(iii) The 9-item School-Burnout Inventory (SBI) [46] will be applied to measure symptoms of school burnout. It has been shown that school burnout predicts subsequent depressive symptoms [47] and that low levels of physical activity are associated with increased school burnout among adolescents [48]. The SBI consists of 10 items and is a multifaceted instrument with three subscales: (i) exhaustion at school; (ii) cynicism towards the meaning of school; and (iii) sense of inadequacy at school. Answers are given on a 5-point Likert-scale ranging from 1 (never) to 5 (always). Evidence in support of the factorial and construct validity of the SBI can be found in the literature [46, 47, 49, 50].

#### Parasitological examinations

In order to determine the prevalence of various intestinal parasites, both stool and urine samples will be collected from each participant. The samples will be subjected to a suite of standardised, quality-controlled diagnostic work-up [51, 52].

(i) A single stool sample will be collected from each child and analysed on the same day. The procedures are as follows. Each student will be given a container, labelled with a unique identification number, in which they will be asked to deposit a stool sample of not more than half the container’s size with their own stool at home and bring it to the school for collection the following morning. In a first step, stool sample (at least 15 g) will be visually examined for the presence of *Taenia* spp. proglottids as well as signs of blood, mucus and diarrhoea. Second, duplicate 41.7 mg Kato-Katz thick smears will be prepared from each stool sample [53]. Slides will be allowed to clear for 30–45 min before being examined under a microscope by experienced laboratory technicians. The number of helminth eggs will be counted and recorded for each species separately. Helminth egg counts will be multiplied by a factor 24 to obtain a proxy for infection intensity, as expressed by the number of eggs per 1 g of stool (EPG) [51, 54]. Possible helminth species to be detected include the three main species of soil-transmitted helminths (i.e. *Ascaris lumbricoides*, hookworm and *Trichuris trichiura*), *Fasciola hepatica* and *Schistosoma mansoni*. The presence of other helminth eggs will be noted, but not quantified.
(ii) For the detection of *Cryptosporidium* spp. and *Giardia intestinalis*, a Crypto-Giardia Duo-Strip® rapid diagnostic test (RDT) (CORIS, BioConcept; Gembloux, Belgium) will be performed on a stool sample, which has been diluted with a commercial buffer [55].
(iii) For the detection of *Helicobacter pylori*, a Pylori-Strip® RDT (CORIS, BioConcept; Gembloux, Belgium) will be performed on a stool sample, which has been diluted with a commercial buffer.
(iv) A single urine sample will be collected from each child. All children will be given a container, labelled with a unique identification number, which they will be asked to fill up full with their urine. Distribution and collection of filled containers will occur on the same day. Each sample will be analysed visually for macrohaematuria and tested with Hemastix® strips to detect blood in urine as a proxy for *Schistosoma haematobium*. A point-of-care circulating cathodic antigen (POC-CCA) urine casette test (Rapid Medical Diagnostics; Cape Town, South Africa) will be used to detect the presence of *S. mansoni* infections [56].
(v) For quality control, a random sample of 10 % of all Kato-Katz and urine filtration slides will be re-examined by a senior technician [57]. In case of discordant results, the slides will be read a third time and results discussed among the technicians until agreement has been reached.

### Data collection and management

The type of data to be collected include: (i) quantitative data on the prevalence of intestinal parasites, measurements of blood pressure and glycated hemoglobin level, anthropometry and the level of physical fitness, cognitive performance and psychosocial health; (ii) socioeconomic status and demographic data, including the geographical location (latitude and longitude expressed in decimal degrees) of the students’ households; and (iii) qualitative data on the feasibility and acceptability of the intervention measures implemented via focus-group discussions.

Data will be double-entered, cross-checked with EpiData 3.1 (EpiData Association; Odense, Denmark) and merged into a single database using STATA version 13.0 (STATA Corp.; College Station, TX, USA).

### Data analysis

The primary objectives of the statistical analysis will be to assess (i) the prevalences of infections and conditions, and their associations with physical fitness, nutritional status, cognitive performance and psychosocial health at baseline and over time; and (ii) the effects of interventions on disease status and other health parameters. The secondary objective will be to assess the feasibility and acceptability of the health interventions implemented.

Parasitological status will be assessed in terms of prevalence and intensity of infection with specific parasite species and the extent of multiparasitism. Clinical and anthropometric indicators, physical fitness, cognitive performance and psychosocial health scores will be characterised by their mean and standard deviation if they are normally distributed and by their median and interquartile range otherwise. Questionnaire data pertaining to the psychosocial health will be expressed as percentages. All indicators will be compared between physically fit/non-infected and physically unfit/infected children and between intervention and control schools.

To assess the effects of the different interventions on the parasitological status, clinical and anthropometric indicators, physical fitness, cognitive performance and psychosocial health, the following statistical procedures will be employed:

(i) Mixed logistic regression models with random intercepts for schools will be used to compare binary data, such as parasitological status and clinical indicators, between the intervention and control groups.
(ii) Linear mixed models with random intercepts for schools will be used for numeric data, such as anthropometric measurements, physical fitness, cognitive performance, and psychosocial health scores and haemoglobin concentration measurements.
  These models will include sex and age of the child, socioeconomic status of the parents or health status or fitness at the baseline survey as well as variables which were not perfectly randomised and might therefore act as confounders. Moreover, as intervention effects might depend on baseline characteristics of the child, stratified analyses and analyses involving interaction terms will be performed. The potential effect modifiers tested include sex, age, socioeconomic status of the parents or health status or fitness at the baseline survey.

### Ethical approval and considerations

Ethical approval for the study has been obtained from the Ethics Committee Northwest and Central Switzerland (EKNZ) in Basel, Switzerland (reference no. 2014–179; obtained on 1 August 2014), and the following ethics committees in Port Elizabeth, South Africa:

(i) NMMU Human Ethics Committee (Human) (reference no. H14-HEA-HMS-002; obtained on 4 July 2014);
(ii) Eastern Cape Department of Education (obtained on 13 August 2014); and
(iii) Eastern Cape Department of Health (obtained on 7 November 2014).

Besides obtaining written informed consent, confidentiality of the study participants will be ensured by giving each participant a unique project-ID number so that all collected data of the schoolchildren will remain anonymous. Data will be used exclusively for scientific research and samples will be discarded after laboratory analyses have been completed. Paper records of the study are kept in locked cupboards in South Africa, accessible only by the main investigators. After 5 years, these records will be destroyed. Data entered into computerised files will be accessible only to authorised investigators or medical personnel directly involved with the study. At the end of the project, successful and appropriate interventions will be provided to the control schools so that the whole community can benefit from this project.

## Discussion

The baseline survey of this study has been conducted in February-March 2015. Subsequently, eight schools, with approximately 1000 schoolchildren, were selected for the study. During this first survey time point, several challenges were met and needed to be addressed. First, the study is conducted in impoverished and harsh environments, where illiteracy and violence are common [58, 59]. In these challenging socioeconomic conditions, the recruited schoolchildren may often be subjected to the lack of sufficient care or neglect by their parents [60–62]. As such, it was difficult to obtain support and written informed consent from the parents/guardians even if the schoolchildren have provided their oral assent. To address this issue, we prepared and conducted several pre-study workshops in the selected schools. The study purposes were explained in detail to the school principals, teachers and parents/guardians, in order to garner their strong support. We also adapted our study as much as possible in response to ideas voiced by teachers and parents to tailor the study further to the needs of the people concerned. Second, three languages, namely Afrikaans, Xhosa and English, are being spoken by the communities in the study area. For example, certain schoolchildren might prefer to speak and write in English, while others from the same school prefer Afrikaans. In addition, Xhosa-speaking children often preferred the tests to be administered in English, with explanations in Xhosa. This proved to be challenging when questionnaires were administered. Furthermore, although the questionnaires were pre-tested with some schoolchildren, the content of the questionnaires employed, particularly the ones focusing on psychosocial health indicators, did not fully match the educational level of the schoolchildren, making it hard for them to understand and answer the questions. To address these issues, we employed native speakers to perform the translation and to pre-test the translated questionnaires among teachers and students before the start of the study. During the actual administration of the questionnaires, we had the help of teachers and school volunteers to explain the questions to the children in their preferred language.

During our sample size calculation, we accounted for a 10 % loss to follow-up. Moving forward with the follow-ups and second phase of intervention, we might expect a more substantial loss (30-40 %) to follow-up as people show considerable mobility in this setting. We will address the potential bias resulting from differential loss to follow-up using inverse probability weighting, i.e. by assigning each follow-up participant the inverse of their prior probability of participation in the follow-up as weight in the follow-up analyses. In addition, multiple imputation will be used to deal with missing data where appropriate. However, it is difficult to predict the extent of the movement of people and hence, our presumed loss to follow-up might not be accurate.

Based on the obtained results, we have designed the health interventions together with local principals and teachers. Such collaboration is aimed at bolstering a sense of community ownership and empowerment, where the community feels that they are taking steps to improve the health of their children. We hope that this approach will further encourage the continued participation of the children and their parents/guardians during the study and sustained use of the interventions after the study has ceased.

In conclusion, the DASH study described here will provide a snapshot on the status of intestinal parasite infections and risk factors for diabetes and hypertension in selected disadvantaged primary schools in Port Elizabeth, South Africa. To our knowledge, such data are currently not available, and hence, our study fills an important void and generates new local evidence. By linking children’s parasitic infection status with the physical fitness, nutritional status, cognitive performance and psychosocial health, this wealth of information will help shed new light on the health consequences incurred by the children and provide guidance for further health interventions in this area. Implementation of setting-specific interventions within the longitudinal study will further highlight the feasibility and scalability of these health interventions in the study area.
